# Identification of isoflavones in the extract of supplements for menopause symptoms by direct infusion electrospray ionization tandem mass spectrometry

**DOI:** 10.1002/ansa.202000013

**Published:** 2020-05-22

**Authors:** Monika Beszterda, Rafał Frański

**Affiliations:** ^1^ Department of Food Biochemistry and Analysis Poznań University of Life Sciences Poznań Poland; ^2^ Faculty of Chemistry Adam Mickiewicz University Poznań Poland

**Keywords:** dietary supplements, fragmentation pattern, negative ion mode, phytoestrogens

## Abstract

In recent years high consumption of dietary supplements has been observed. However, the consumption of dietary supplements may lead to the unexpected side effects that can be related to the number of adulterated supplements quite often marketed. It has prompted the search for a fast and reliable method of identification of main active compounds in the supplements. In this study, the isoflavones present in the methanol extracts of dietary supplements for menopause symptoms were identified by using direct infusion electrospray ionization tandem mass spectrometry. The product ion spectra of [M‐H]^−^ ions derived from the extracts matched very well those obtained for standard compounds. Daidzein, genistein, and glycitein were identified in the soy‐based supplements, while daidzein, formononetin, and biochanin A were identified in the red clover‐based ones. The respective [M+Na]^+^ ions were also detected; however, their product ion spectra did not allow isoflavone identification. It can be concluded that the main isoflavones present in the extracts of dietary supplements can be successfully and quickly identified by using the direct infusion electrospray ionization in negative ion mode, followed by the tandem mass spectrometry experiment.

## 1 INTRODUCTION

It is well known that for the analysis of particular compounds in the complex matrices (e.g. food samples) the liquid chromatography/electrospray ionization mass spectrometry (LC/ESI‐MS) seems to be the most suitable method. However, there are a number of examples that illustrate that for qualitative analysis of the compounds present in food samples the direct infusion/electrospray ionization mass spectrometry (DI/ESI‐MS) can also be successful. For example, DI/ESI‐MS can be reliable for the assessment of adulteration of oils,^1^, ^2^, ^3^, ^4^, ^5^ whisky,^6^ cachaça,^7^, ^8^ arabica coffee,^9^ or for quality control of wine.^10^ Flavonoids are among the compounds that have been successfully identified by DI/ESI‐MS, for example, in wine,^11^ berries,^12^, ^13^ pequi,^14^ and rose species.^15^

Isoflavones are a subclass of flavonoids and the most common phytoestrogens.^16^, ^17^ Biological activities of isoflavones present in dietary supplements have been widely studied,^18^, ^19^, ^20^ for example, isoflavone impact on the health of postmenopausal women.^21^, ^22^ Isoflavones prevent bone resorption,^23^, ^24^, ^25^ moreover they increase the efficacy and prevent the adverse effects of cancer chemotherapy and radiation therapy.^26^, ^27^ Of course, LC/ESI‐MS has been successfully used for analysis of isoflavones in dietary supplements.^28^, ^29^, ^30^

In this work, it is demonstrated, to the best of our knowledge for the first time, that DI/ESI‐MS can be also successfully used for qualitative analysis of isoflavones in the extract of dietary supplements. In order to unequivocally identify the isoflavones, tandem mass spectrometry experiments and accurate mass measurements have been performed. Furthermore, it has been demonstrated that the presence of abundant interferences (isobaric compounds) does not disturb identification of isoflavones. It may be argued that qualitative identification of isoflavones in dietary supplements, without their quantitative analysis is out of importance. However, quantitative analysis always has to be preceded by reliable qualitative identification, whereas the qualitative identification of isoflavones is sometimes disputable.^31^ ^,^ ^32^

There is a high risk that the compounds present in the analyzed mixture in low concentrations or characterized by low ESI response will not be detected upon DI/ESI‐MS analysis. On the other hand, DI/ESI‐MS is definitely less time consuming than LC/ESI‐MS. Therefore, for fast screening of a vast number of samples or for fast qualitative identification of the compounds of main interest in the analyzed samples, the DI/ESI‐MS may be an attractive choice. Furthermore, it seems to be likely that DI/ESI‐MS can be also successfully used for quality control of the supplements.

## 2 MATERIALS AND METHODS

All analyzed samples were commercial food supplements for menopausal symptoms in intact retail packaging which were collected by the authors. The supplements based on soy (**1**, **2**, **3**) or red clover (**4**, **5**, **6**) were purchased from a local pharmacy and were from various batches. Detailed characterization of the preparations is given in the Table 1.

**TABLE 1:** Characteristics of the analyzed food supplements

No.	Source of isoflavones	Other active ingredients	Form
1	Soy (*Glycine max*) extract	Extract from hop cones, d‐alpha‐tocopheryl succinate, ground flaxseed, cholecalciferol, pyridoxine hydrochloride	Tablets
2	Soy (*Glycine max*) extract	–	Capsules
3	Soy (*Glycine max*) extract	–	Tablets
4	Red clover (*Trifolium pratense* L.) extract	Extract from hop cones	Tablets
5	Red clover (*Trifolium pratense* L.) extract	l‐Ascorbic acid, cholecalciferol, chromium (III) chloride, pyridoxine hydrochloride	Tablets
6	Red clover (*Trifolium pratense* L.) extract	l‐Tryptophan, saffron flower (*Crocus sativus*) extract, pyridoxine hydrochloride, folic acid, cyanocobalamin	Capsules

The capsule content (100 mg from 350 mg of fine powder) was weighed and dissolved in 1 mL of pure methanol. The sample was shaken at 500 rpm for 30 min (Vortex 3, IKA‐Werke GmbH, Germany), sonicated, and filtered through syringe filters with a pore size of 0.45 μm (Macherey‐Nagel GmbH, Germany). The extracts were stored at 5°C. Prior to the DI/ESI‐MS analysis, the sample was further diluted 1:100 in pure methanol. Standards of isoflavones (daidzein, genistein, glycitein, formononetin, biochanin A, purity > 98 %) were obtained from Sigma–Aldrich (Poznań, Poland) and used without purification.

The DI/ESI‐MS analyses were performed on a Waters/Micromass (Manchester, UK) Q‐TOF Premier mass spectrometer (software MassLynx v. 4.1, Manchester, UK). The sample solutions were infused into the ESI source by a syringe pump at a flow rate of 5 μL/min. The electrospray voltage was 2.7 kV and the cone voltage −30 V. The source temperature was 80°C and the desolvation temperature was 250°C. Nitrogen was used as the cone gas and desolvating gas at the flow rates of 0.8 and 13 L/min, respectively. Argon was used as a collision gas at a flow rate 0.5 mL/min in the T‐wave collision cell. This flow rate resulted in the collision cell pressure of 0.3 Pa. The applied collision energy (CE, laboratory frame), the most important parameter for collision induced dissociation tandem mass spectrometry experiments (CID‐MS/MS), is indicated in the captions to the product ion spectra shown. In order to perform the accurate mass measurement, margaric acid has been added to the analyzed extract (concentration 10^−5^ mol/dm^3^) and its [M‐H]^−^ ion (C_16_H_33_COO^−^, exact *m/z* 269.2480) was used as a lock mass.

## 3 RESULTS AND DISCUSSION

**FIGURE 1:**
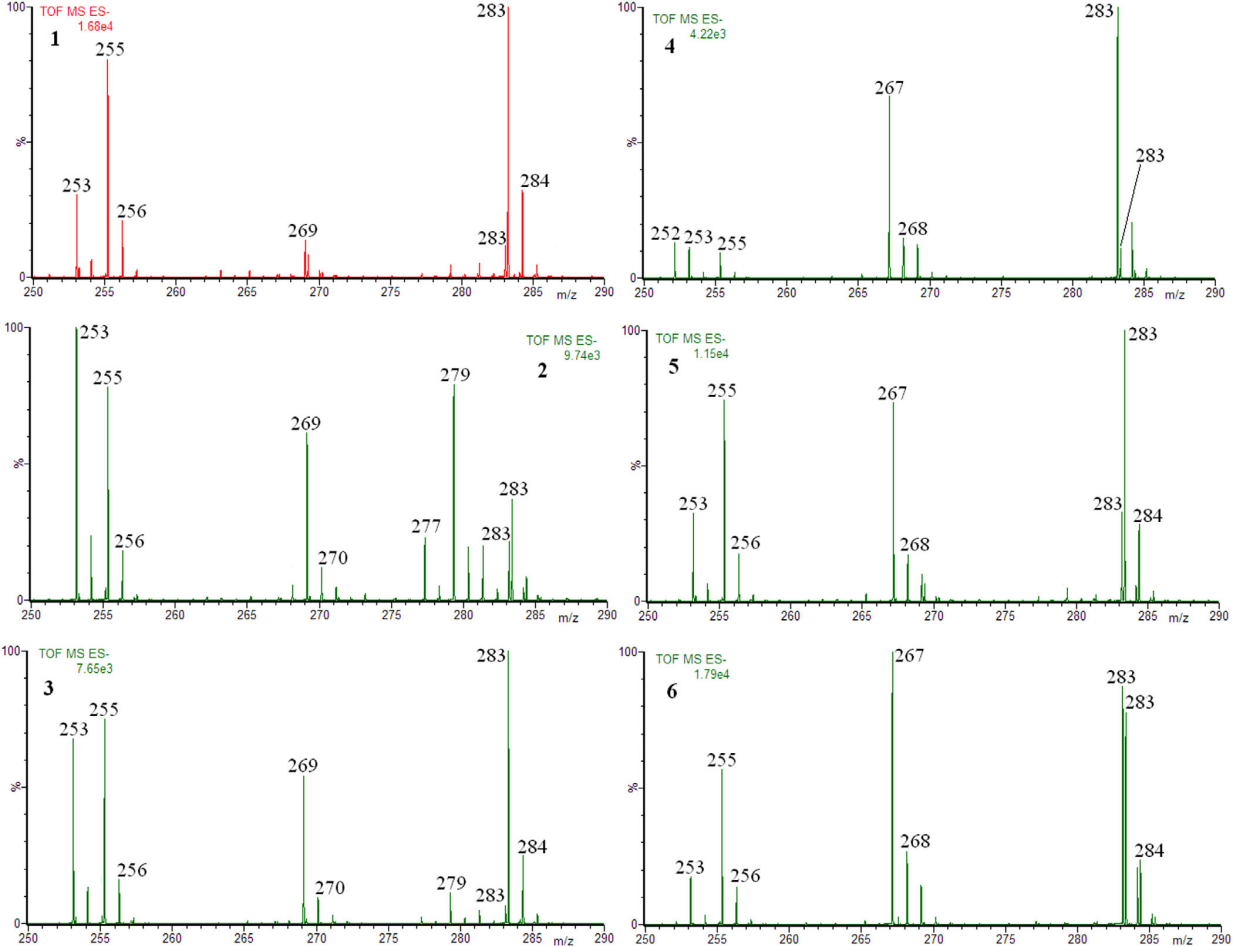
The mass spectra, in *m/z* range 250‐290, obtained for the extracts of soy based supplements (left column, **1**, **2**, and **3**) and red clover based supplements (right column, **4**, **5**, and **6**)

We found that the signals that could derive from isoflavones are in the range of 250‐290 *m/z*, therefore the mass spectra of the supplement extracts in the range of 250‐290 are shown in Figure 1 (in the Supporting Information, there are full scan mass spectra in the *m/z* range 100‐1000 of extracts of supplements **1** and **5**, as a representative example, Figure S1).

It is reasonable that the abundant ions at *m/z* 255 and 283 correspond to the [M‐H]^−^ ions of the most common fatty acids, namely palmitic and stearic acids, respectively. The expected [M‐H]^−^ ions of isoflavones are at *m/z* 253 (e.g. daidzein), 267 (e.g. formononetin), 269 (e.g. genistein), and 283 (e.g. glycitein or biochanin A). The ion [M‐H]^−^ of stearic acid and the ion [M‐H]^−^ of glycitein/biochanin A have the same nominal *m/z* values, namely 283. The exact mass of the former is slightly higher than the exact mass of the latter, thus it is clear which ions at *m/z* 283 may correspond to glycitein/biochanin A and which to stearic acid (e.g. the low abundant ion at *m/z* 283 for extracts **1** and **3** corresponds to glycitein, whereas the highly abundant ion at *m/z* 283 corresponds to stearic acid, Figure 1).

**FIGURE 2:**
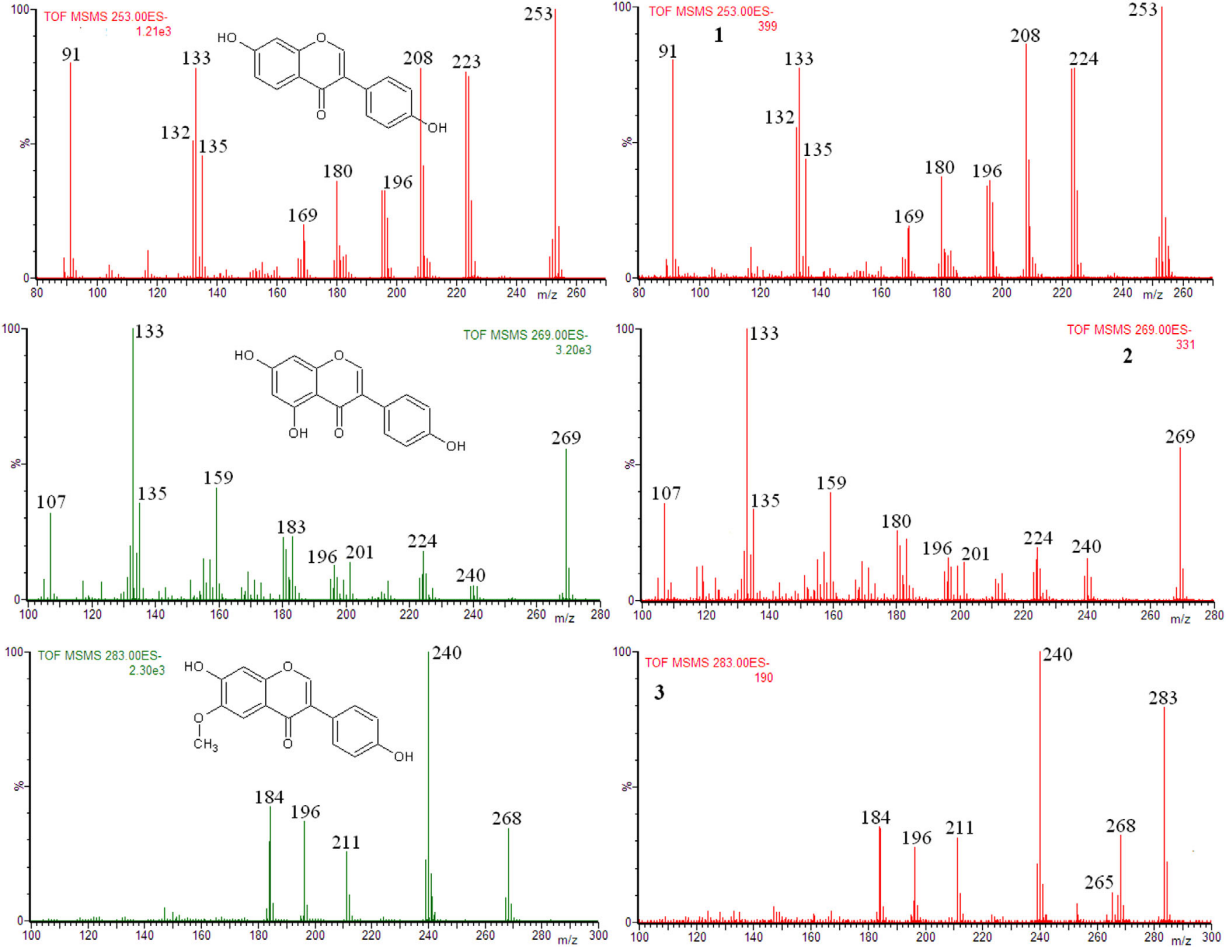
Left column: product ion spectra of [M‐H]^−^ ions of standards: daidzein (*m/z* 253), genistein (*m/z* 269), and glycitein (*m/z* 283). Right column: product ion spectra of ions at m/z 253, 269, and 283 obtained for the exctracts **1** , **2**, and **3**, respectively (CE = 30 eV)

The obtained product ion spectra of the ion at *m/z* 253, 269, and 283 and the product ion spectra of [M‐H]^−^ ion of daidzein, genistein, and glycitein standards, confirmed the presence of these isoflavones in the soy based supplements (extracts **1**, **2**, and **3**) as shown in Figure 2 and in the Supporting Information (Figures S2‐S4).

The product ion spectra shown in Figure 2 were obtained at the collision energy 30 eV. The identical, characteristic fragmentation patterns obtained for [M‐H]^−^ ions of daidzein, genistein, and glycitein standards and for ions at *m/z* 253, 269, and 283 of the analyzed extracts permit drawing a conclusion that these ions of the analyzed extracts undoubtedly correspond to the [M‐H]^−^ ions of daidzein, genistein, and glycitein, respectively.

The first step of the fragmentation of [M‐H]^−^ ions of methoxylated flavonoids is the loss of the methyl radical, thus the formation of the [M‐H‐CH_3_]^−•^ product ions.^31^ At the collision energy 30 eV, in the product ion spectrum of [M‐H]^−^ ion of glycitein standard there is no signal at *m/z* 283 (ion [M‐H]^−^ of glycitein). As expected, the product ion [M‐H‐CH_3_]^−•^ is observed at *m/z* 268 and the subsequent decomposition of ion [M‐H‐CH_3_]^−•^ has yielded a characteristic fragmentation pattern (Figure 2). Identical fragmentation patterns were obtained for the ion at *m/z* 283 of extracts **1**, **2**, and **3**, thus glycitein is present in these extracts. In the product ion spectra of the ions at *m/z* 283 of extracts **1**‐**3**, there are signals at *m/z* 283, which undoubtedly correspond to the [M‐H]^−^ ions of stearic acid (Figure 2 and Figure S4). The fragmentation of the [M‐H]^−^ ions of saturated fatty acids is poor and consists mainly in the loss of H_2_O.^34^, ^35^, ^36^ And indeed, in the mass spectra of extracts **1** and **3** the signals of ions [M‐H‐H_2_O]^−^ at *m/z* 265 are clearly seen (Figure 2 and Figure S4). For extract **2**, the ion [M‐H]^−^ of stearic acid has low abundance (Figure 1), therefore, its product ion [M‐H‐H_2_O]^−^ was not observed (Figure S4).

It is worth adding that the mass spectra shown in Figure 2 are in good agreement with those of daidzein and genistein presented by Vessecchi et al.^33^

It is well known that product ion spectra of isomers can be very similar or even identical. Furthermore, the product ion spectra obtained at an interval of a few weeks (under identical instrumental conditions) may differ in relative ion abundances because of the fluctuations in pressure inside the mass spectrometer.^37^ Among the isomers of glycitein, isoprunetin has the fragmentation pattern most similar to that of glycitein.^31^ Therefore, we obtained the product ion spectrum of [M‐H]^−^ of isoprunetin, as shown in Supporting Information (Figure S5), whose fragmentation pattern is different than that of glycitein.

**FIGURE 3:**
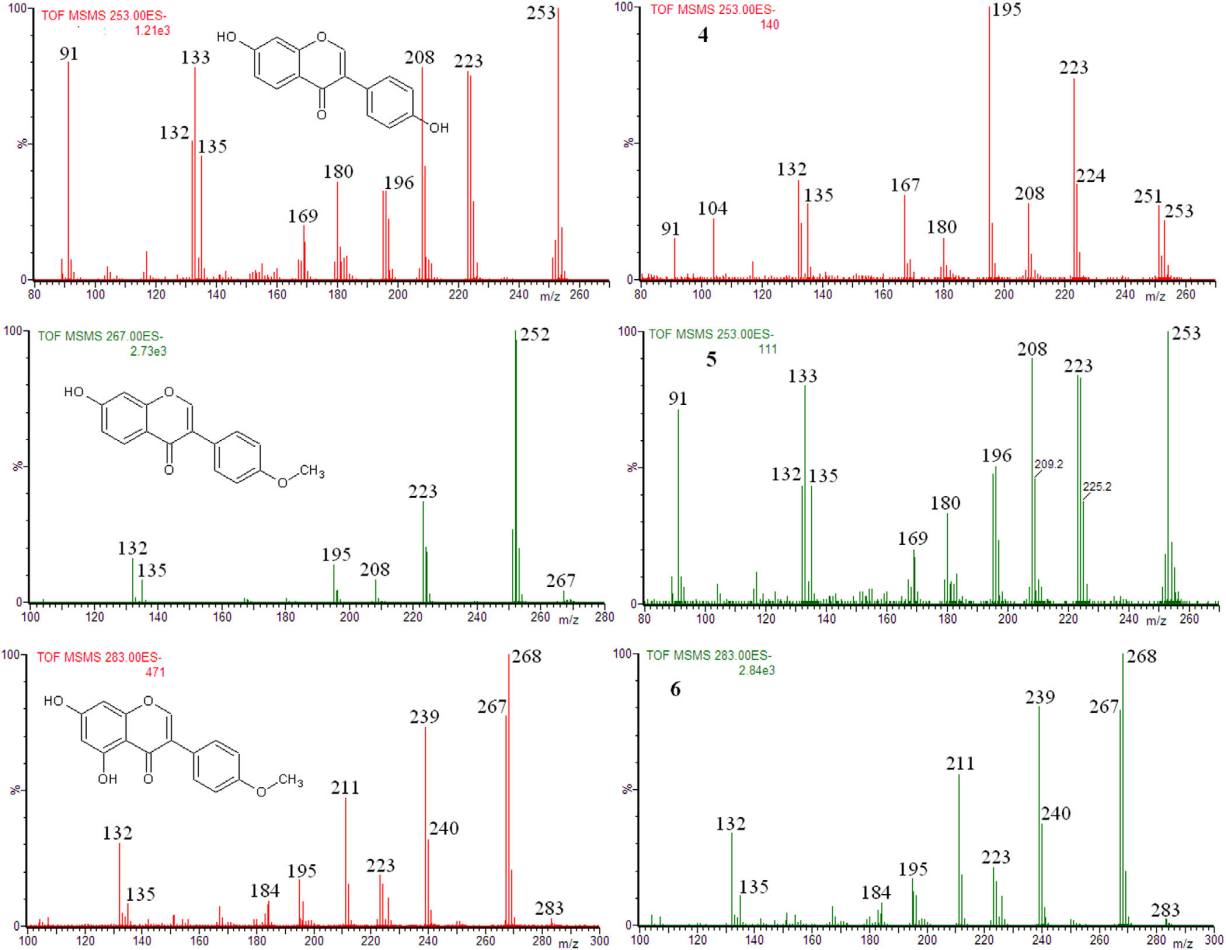
Left column: product ion spectra of [M‐H]^−^ ions of standards: daidzein (*m/z* 253), formononetin (*m/z* 267), and biochanin A (*m/z* 283). Right column: ions product ion spectra of ions at *m/z* 253, 267, and 283 obtained for the extracts **4**, **5**, and **6**, respectively (CE = 30 eV)

The ion at *m/z* 253 of extracts **5** and **6** corresponds to daidzein (analogously as for the extracts of soy based supplements) as shown in Figure S6. However, the presence of daidzein in extract **4** cannot be definitely confirmed or excluded, as shown in Figure 3. It is clear that the ions (eg, at *m/z* 252, or at *m/z* 253 other than [M‐H]^−^ of daidzein) affect the obtained product ion spectrum.

A comparison of the obtained product ion spectra of the ion at *m/z* 267 and 283 and the product ion spectra of [M‐H]^−^ ion of formononetin and biochanin A standards, confirmed the presence of these isoflavones in the red clover based supplements (extracts **4**, **5**, and **6**) as shown in Figure 3 and in Supporting Information (Figures S7 and S8).

It should be added that the fragmentation pattern of prunetin [M‐H]^−^ ion is very similar to that of biochanin [M‐H]^−^ ion.^31^, ^38^ Therefore, it can be argued if the ions at *m/z* 283 really correspond to biochanin A, and not to prunetin. On the other hand, in comparison to the concentration of biochanin A, the concentration of prunetin is very low in red clover.^39^ Therefore, it can be taken for granted that in extracts **4**, **5**, and **6** we deal with biochanin A.

**FIGURE 4:**
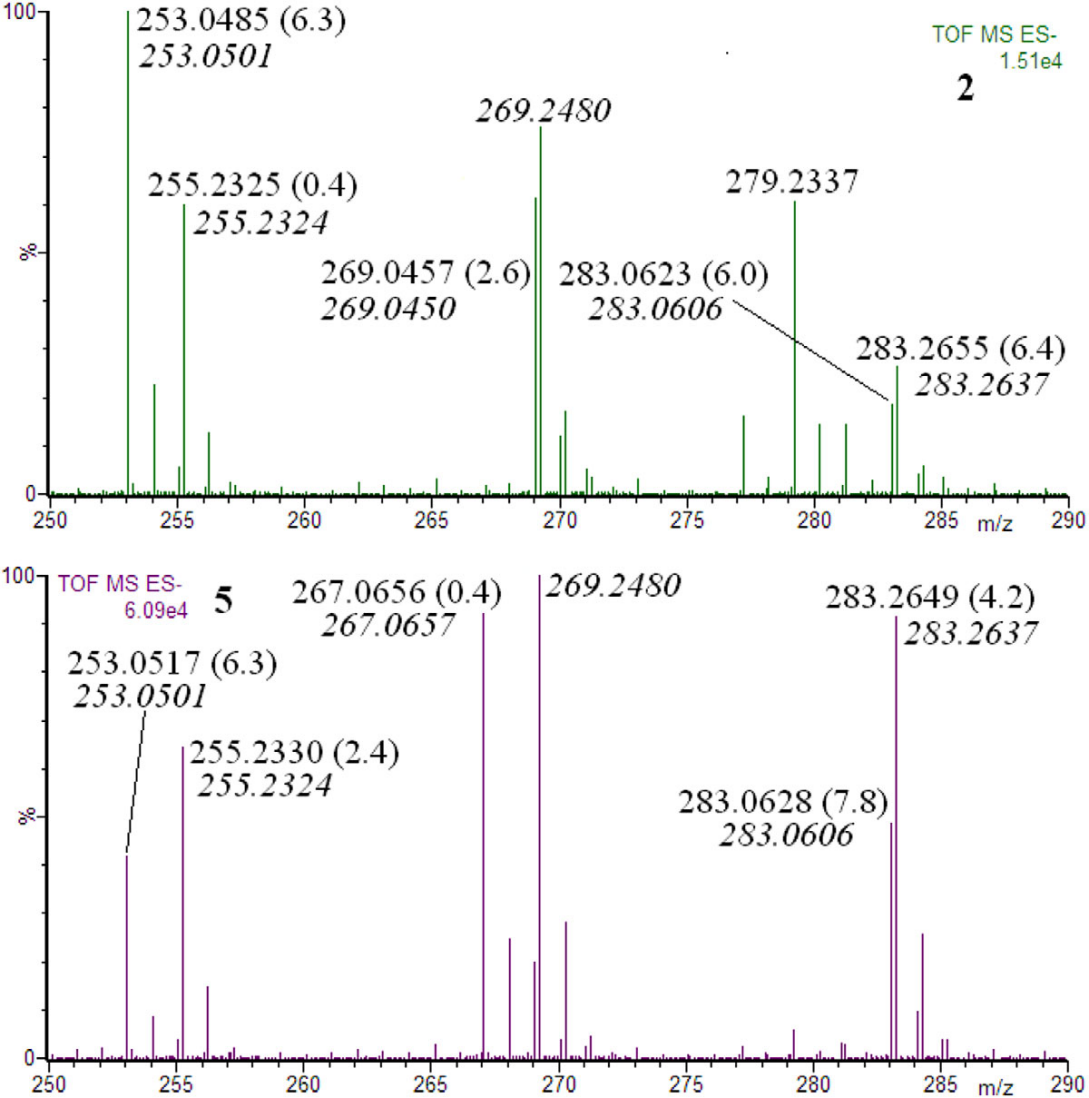
The exemplary results of accurate mass measurements. Italic type, calculated exact masses; normal type, measured accurate masses; in brackets, errors (ppm)

In order to confirm the elemental composition of the detected ions, their accurate masses were measured. Margaric acid was added to the extracts and its [M‐H]^−^ ion was used as a lock mass (*m/z* 269.2480). Exemplary results are shown in Figure 4.

As shown in Figure 4, the measured accurate masses of [M‐H]^−^ ions of isoflavones (as well as fatty acids) confirmed their elemental compositions. For the identification of the compounds whose isomers are not expected, the accurate mass measurements can be used as a method of their identification (an alternative method to tandem mass spectrometry). However, taking into account the diversity of flavonoids, the accurate mass measurements can be used only as a confirmation.

**FIGURE 5:**
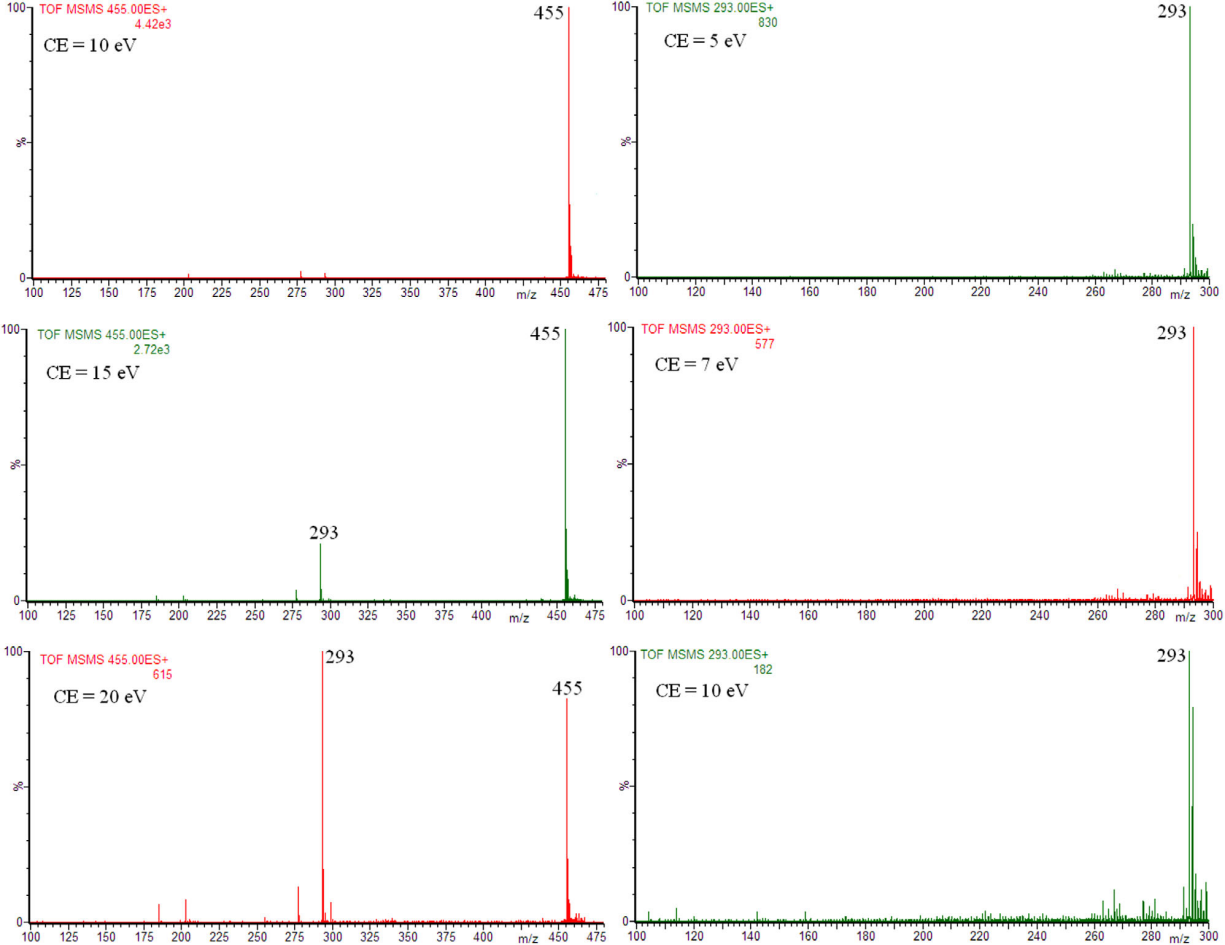
The product ion spectra of [M+Na]^+^ ions of genistein glycoside (*m/z* 455, left column) and genistein (*m/z* 293, right column) obtained for extract **3**. At higher collision energy the dramatic decrease of ion abundances is observed (4.42 × 10^3^→2.72 × 10^3^→615 for genistein glycoside, 830→577→182 for genistein)

We also performed DI/ESI‐MS analysis in the positive ion mode. Sometimes the [M+Na]^+^ ions were detected for both isoflavones and isoflavone glycosides (the presence of sodium is not surprising), exemplary full scan mass spectrum is shown in Supporting Information (Figure S9). On the other hand, the collision induced dissociation (CID) of [M+Na]^+^ ions did not allow isoflavone identification. Figure 5 shows the product ion spectra of the ion at *m/z* 455, which is most probably the [M+Na]^+^ ion of genistein glycoside (molecular weight 432) and the product ion spectra of the ion at *m/z* 293, which is most probably the [M+Na]^+^ ion of genistein.

For the [M+Na]^+^ ion of genistein glycoside, at higher collision energy a product ion formed as a result of glucose moiety loss (the loss of mass 162) is observed. In Figure S10, the product ion spectrum of [M+Na]^+^ ion of genistein standard is presented. For the [M+Na]^+^ ion of genistein no product ions were formed. At higher collision energy a dramatic decrease in the ion abundances is observed for both [M+Na]^+^ ions. The reasonable explanation of the decrease in these ion abundances is that CID of [M+Na]^+^ ions consist in the loss of Na^+^ ion (which has too low mass to be detected by the majority of mass spectrometers).

The question is why isoflavone glycosides were detected in the positive ion mode and not in the negative one. The plausible explanation is that glycosides, in the negative ion mode, have low ESI response. Glycosides have one acid phenolic group less, since one such group is substituted by sugar moiety. Sugars are definitely weaker acids than phenols, (p*K*a of phenol is 10, whereas p*K*a of glucose is much higher).^40^ Furthermore, glycosides are surely more prone to form complexes with Na^+^ in comparison to free aglycones (oxygen atoms of sugar moiety can participate in bonding Na^+^). Formation of the sodium complexes prevents the formation of [M‐H]^−^ ions.

Isoflavone glycosides were not detected in the analyzed extracts of dietary supplements by using DI/ESI‐MS in the negative ion mode and it can be regarded as a serious limitation of the method, since the bioavailability of glycosides is higher than that of aglycones.^41^ On the other hand, aglycones are faster absorbed than glycosides.^42^, ^43^ Furthermore, from the biological activity point of view, the identification of aglycones is of crucial importance.^16^ It is also clear that aglycones were formed from the respective glycosides (e.g. during supplement production or even during the storage of the extracts).

During the ESI‐MS analysis of flavonoids in the complex samples the matrix effects may lead to the ion suppression/enhancement and it can be a serious problem even in LC/ESI‐MS analysis.^44^, ^45^, ^46^ Nevertheless, for quantitative analysis the matrix effect has to be precisely determined.^47^, ^48^ It is clear that during the DI/ESI‐MS analysis (without separation step), the ion suppression can be a much more serious problem preventing the quantitative determination of the flavonoids.^12^ Of course, the ion suppression may be so high that even qualitative determination can be impossible.^11^ On the other hand, the suppression effect can be negligible in the case of compounds showing very high ESI response, for example, anthocyanins in positive ion mode.^11^ Our findings indicate that suppression effect does not prevent to identify the flavonoids in the extracts of dietary supplements by using DI/ESI‐MS(‐). It is worth adding that Kumar et al. have also found that for the analysis of flavonoids in the extracts of flowers of rose species, the DI/ESI‐MS(‐) is more useful than DI/ESI‐MS(+).^15^

## 4 CONCLUSION

Summing up, the main isoflavones present in the extracts of dietary supplements can be successfully identified by using DI/ESI‐MS in negative ion mode, followed by the tandem mass spectrometry experiment. However, sometimes the obtained results can be ambiguous (Figure 3, extract **5**). On the other hand, the identification of glycitein in extracts **1**‐**3** (Figure 2) or biochanin A in extracts **4**‐**6** (Figure 3), indicates that sometimes the presence of the abundant ion, which has the same nominal *m/z* as the low abundant analyzed ion, does not prevent the identification of the latter. In other words, it seems to be likely that in spite of the presence of interferences (isobaric compounds) the quality control of the supplements by using DI/ESI‐MS may be possible.

By using DI/ESI‐MS in the positive ion mode, the signals of isoflavone glycosides can be detected, however, the identification of isoflavone glycosides or aglycones cannot be performed. In other words, the matrix effect disturbs the flavonoid identification by using DI/ESI‐MS(+) and allow their identification by using DI/ESI‐MS(‐).

## CONFLICT OF INTEREST

The authors declare no conflict of interest.

## Supporting information

Supporting information

## Data Availability

The data that support the findings of this study are available in the Supporting Information of this article.
