# Supplementary material for: Identification of isoflavones in the extract of supplements for menopause symptoms by direct infusion electrospray ionization tandem mass spectrometry
Source: Anal Sci Adv. 2020 May 22;1(3):143–51. doi: 10.1002/ansa.202000013 (PMC10989144; doi:10.1002/ansa.202000013)
Supplement: Supplementary file 1 — Supporting information [file ANSA-1-143-s001.doc]

Identification of isoflavones in the extract of supplements for menopause symptoms by direct infusion electrospray ionization tandem mass spectrometry **– supporting information**


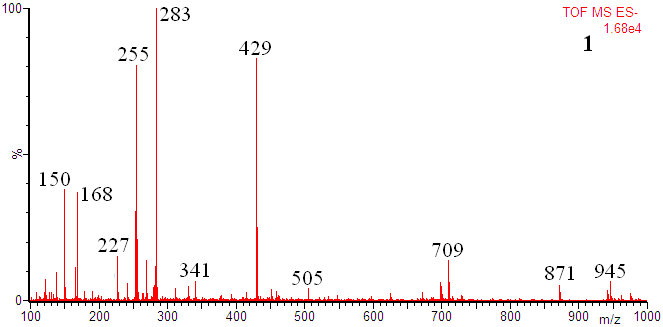

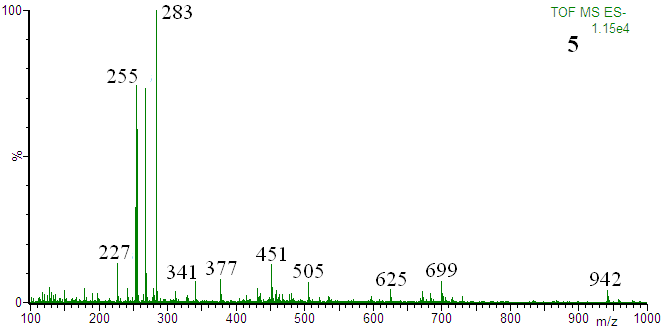


**Figure 1s.** The full scan mass spectra obtained in negative ion mode for the extracts of supplement **1** and **5**.


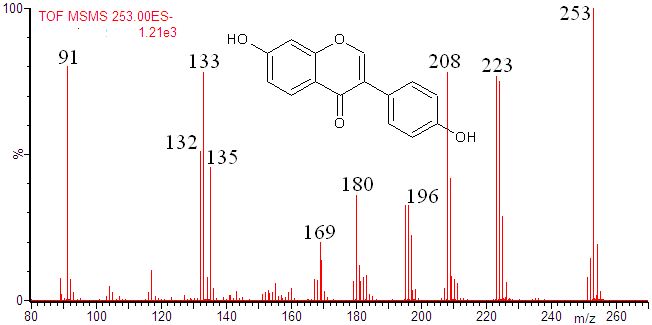


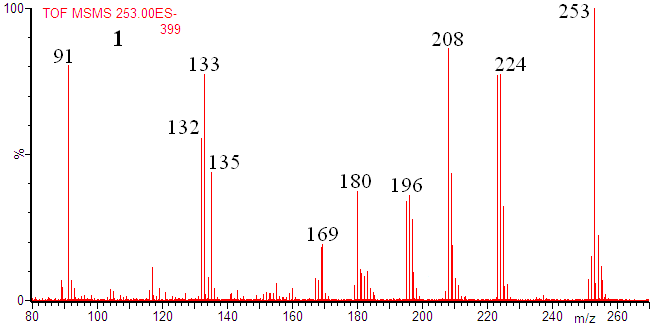


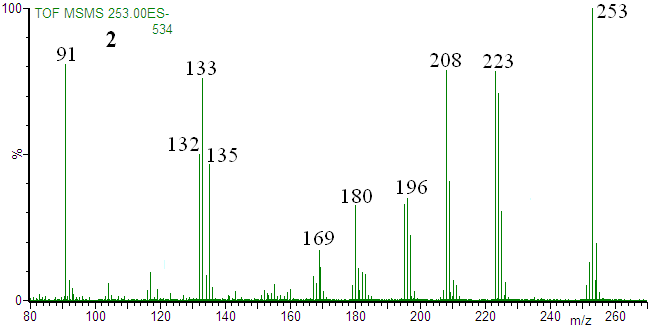


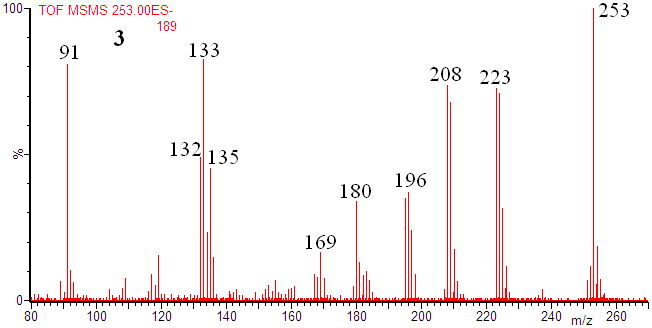


**Figure 2s.** Product ion spectra of [M-H]^-^ ion of daidzein (m/z 253) and ions at m/z 253 obtained for the exctracts **1**, **2** and **3** (CE = 30 eV).


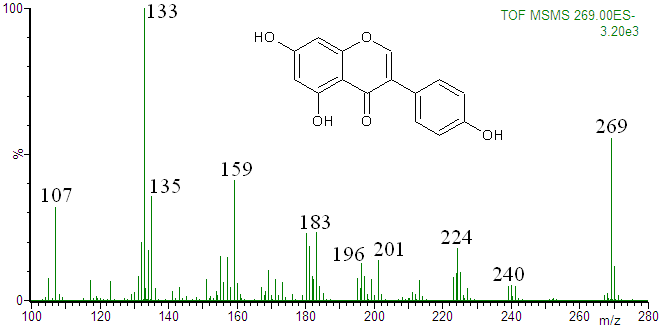


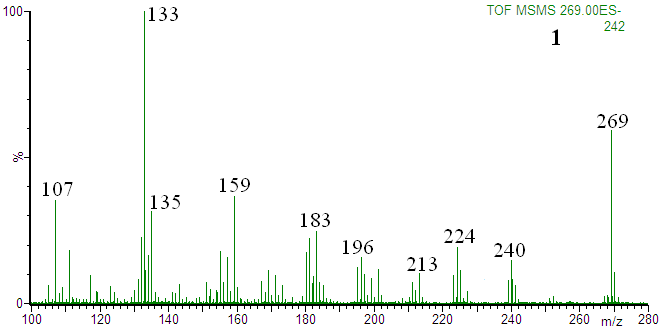


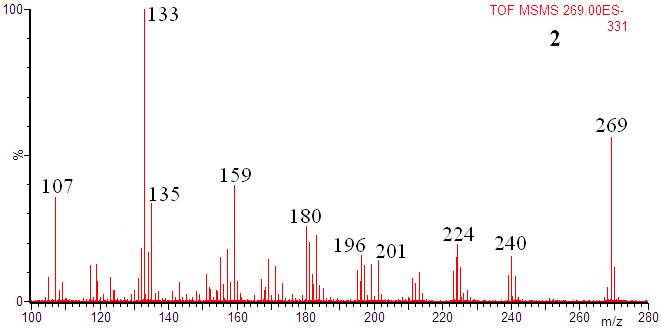


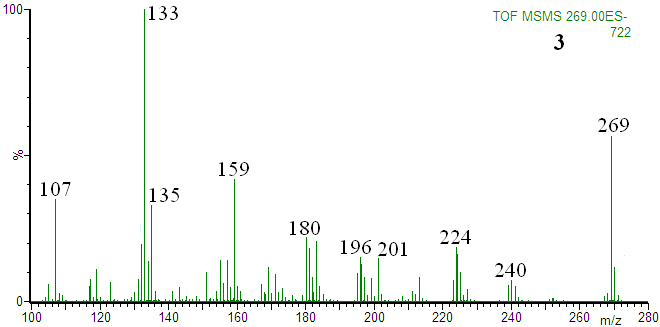


**Figure 3s.** Product ion spectra of [M-H]^-^ ion of genistein (m/z 269) and ions at m/z 269 obtained for the exctracts **1**, **2** and **3** (CE = 30 eV).


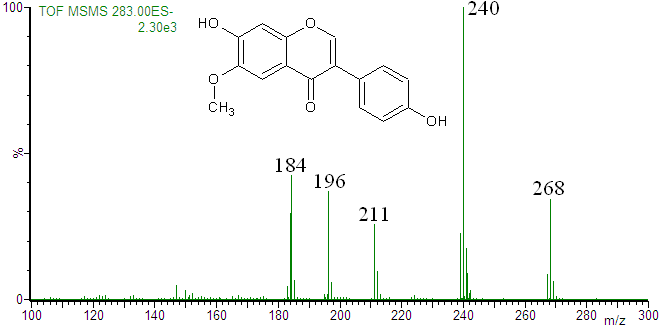


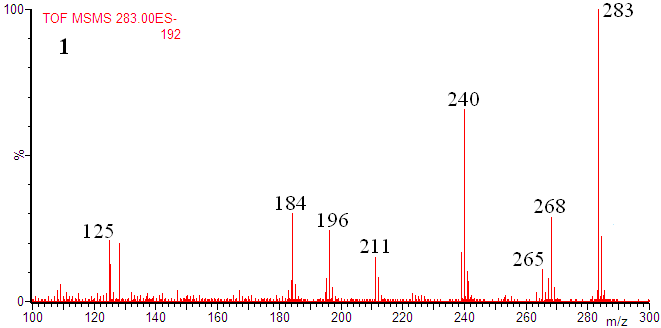


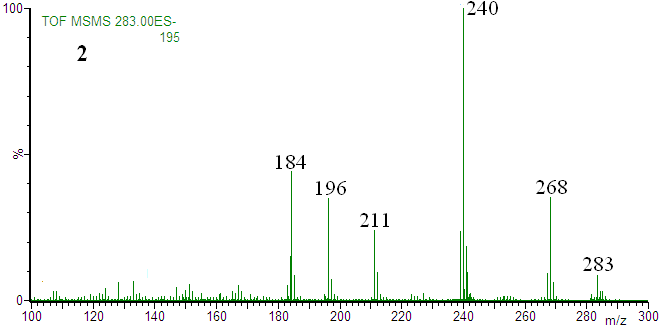


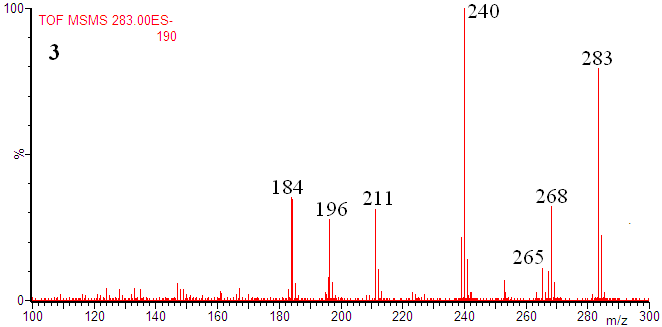


**Figure 4s.** Product ion spectra of [M-H]^-^ ion of glycitein (m/z 283) and ions at m/z 283 obtained for the extracts **1**, **2** and **3** (CE = 30 eV).


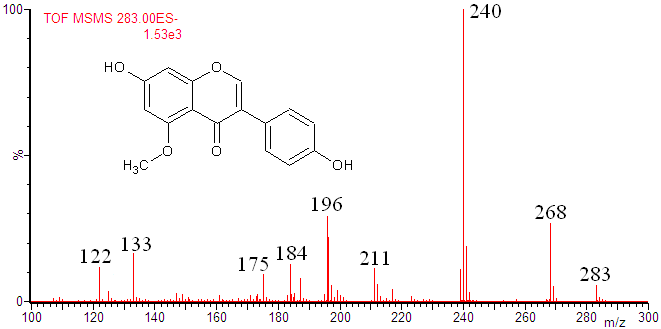


**Figure 5s.** Product ion spectrum of [M-H]^-^ ion of isoprunetin (m/z 283, CE = 30 eV).


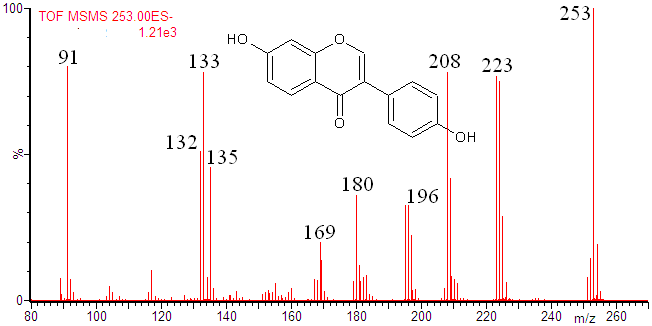


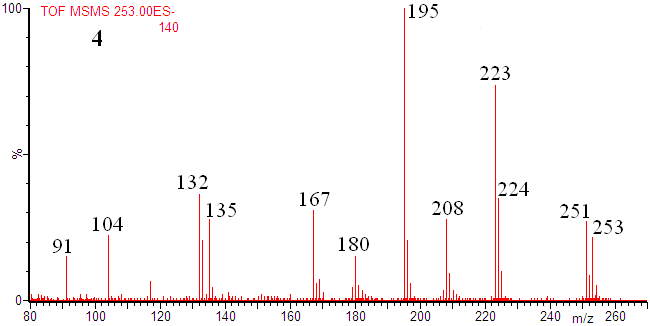


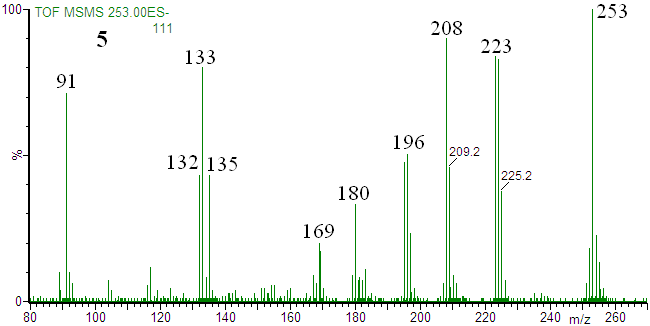


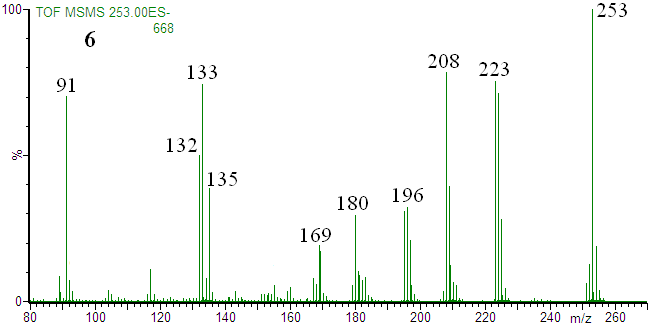


**Figure 6s.** Product ion spectra of [M-H]^-^ ion of daidzein (m/z 253) and ions at m/z 253 obtained for the extracts **4**, **5** and **6** (CE = 30 eV).


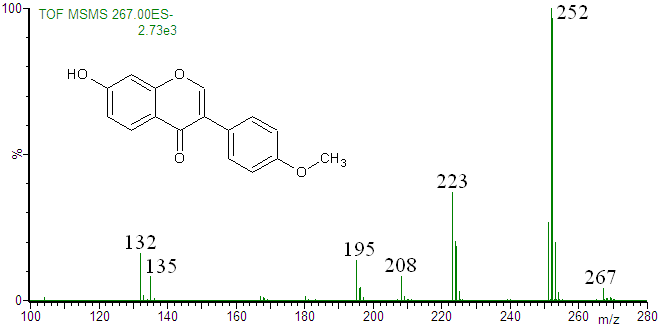


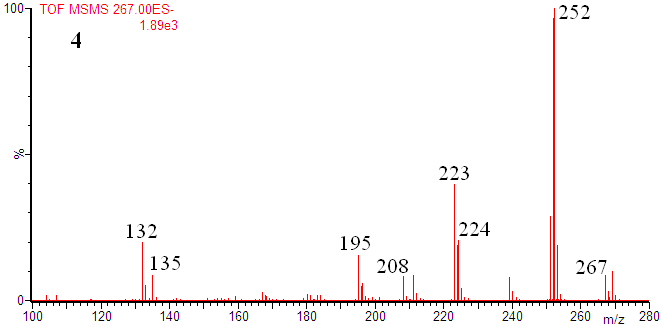


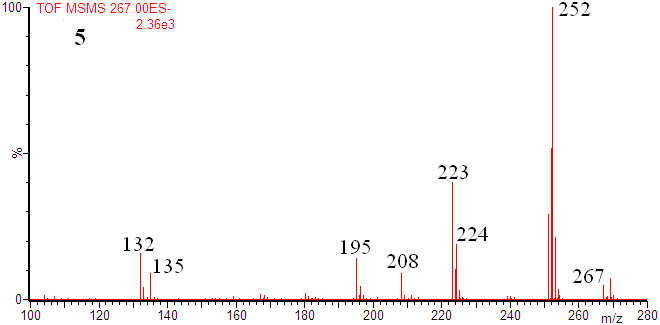


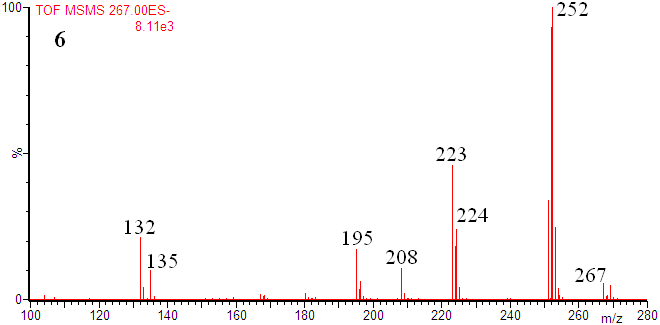


**Figure 7s.** Product ion spectra of [M-H]^-^ ion of formononetin (m/z 267) and ions at m/z 267 obtained for the extracts **4**, **5** and **6** (CE = 25 eV).


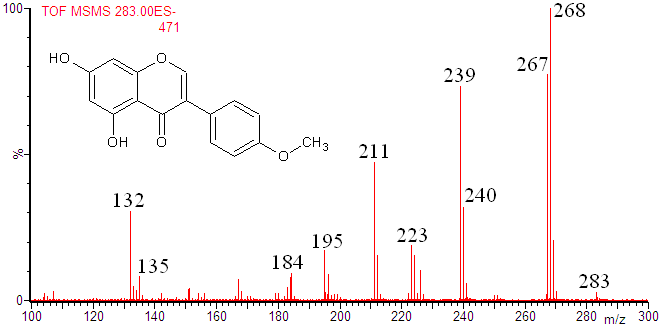


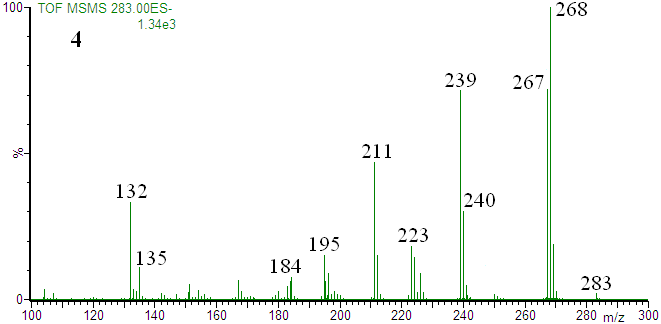


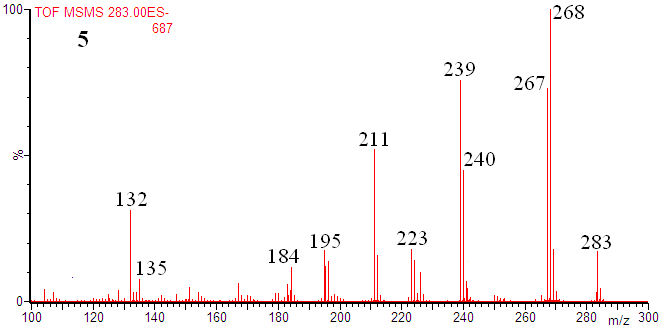


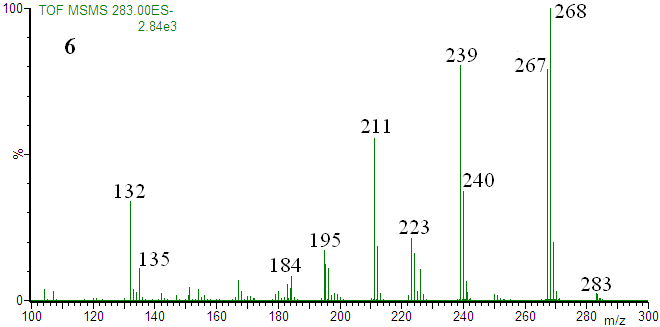


**Figure 8s.** Product ion spectra of [M-H]^-^ ion of biochanin A (m/z 283) and ions at m/z 283 obtained for the extracts **4**, **5** and **6** (CE = 30 eV).


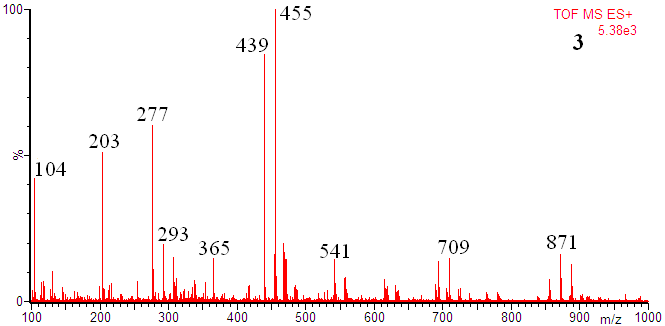


**Figure 9s.** Exemplary full scan mass spectrum of extract **3** obtained in positive ion mode.


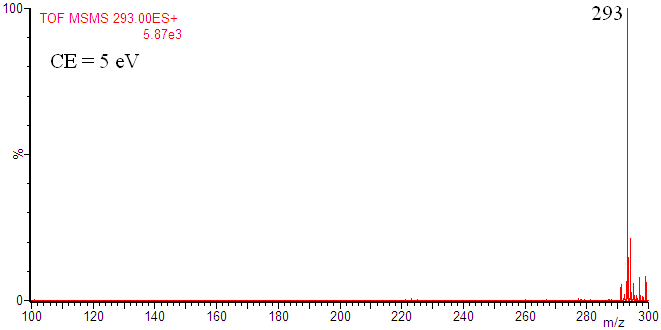


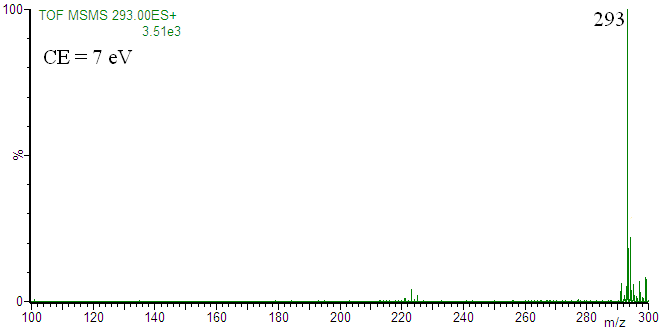


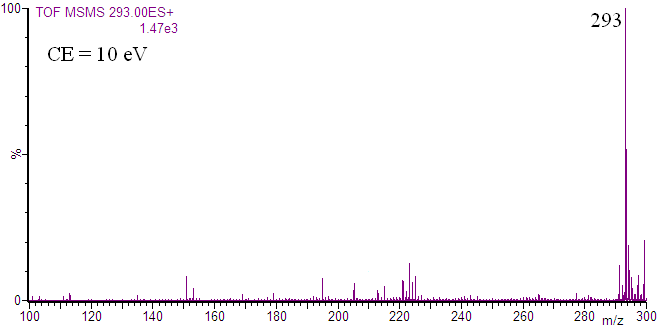


**Figure 10s.** The product ion spectra of [M+Na]^+^ ions of genistein standard (*m/z* 293). At higher collision energy the dramatic decrease of ion abundances is observed (5.87x10^3^→3.51x10^3^→1.47x10^3^).
